# Association between mean corpuscular volume and 3 month outcome in patients with acute ischemic stroke: a second analysis based on a prospective cohort study

**DOI:** 10.3389/fneur.2025.1675822

**Published:** 2025-11-05

**Authors:** Zheng Lv, Yanrong Miao, Meiying Zhu, Fei Li

**Affiliations:** Department of Rehabilitation Medicine, Shenzhen Longgang Central Hospital, Shenzhen Ninth People’s Hospital, Guangzhou University of Chinese Medicine Shenzhen Clinical School of Medicine, Shenzhen, Guangdong, China

**Keywords:** acute ischemic stroke, mean corpuscular volume, prognosis, non-linear relationship, risk stratification

## Abstract

**Introduction:**

Acute ischemic stroke is a major cause of global mortality and disability. This study aimed to investigate the association between mean corpuscular volume (MCV) and 3-month outcomes in acute ischemic stroke patients.

**Methods:**

This study is a secondary analysis based on a prospective cohort study conducted at a single center in Korea from January 2010 to December 2016. The study included 1,906 acute ischemic stroke patients. The exposure variable was MCV measured within 24 hours of admission, and the outcome variable was the modified Rankin Scale (mRS) score at 3 months post-admission. Covariates included age, gender, body mass index, hemoglobin, hematocrit, liver function indicators, diabetes history, and stroke etiology.

**Results:**

Piecewise linear regression analysis revealed a non-linear U‑shaped association of MCV on adverse outcomes, with a critical turning point at 92.1 fl. After full adjustment (Model 3), when MCV was below 92.1 fL, each 1 fL increase was associated with an 8% lower odds of poor 3-month outcome (OR = 0.92, 95% CI: 0.88–0.96, *p* = 0.0003). Conversely, when MCV exceeded 92.1 fL, each 1 fL increase was associated with a 4% higher odds of poor outcome (OR = 1.04, 95% CI: 1.00–1.08, *p* = 0.0391).

**Conclusion:**

There was a U‑shaped association between MCV and three-month outcome in patients with acute ischemic stroke.

## Introduction

Acute ischemic stroke (AIS) is a major cause of death and disability worldwide (1, 2). According to a 2021 study, approximately 12 million people experience a stroke each year globally, with acute ischemic stroke accounting for about 87% of all stroke cases (3). In China, the incidence of acute ischemic stroke is also increasing annually, with data from 2019 indicating that there are about 3 million new stroke cases each year, the majority of which are acute ischemic strokes (4, 5). Three-month outcomes, such as functional recovery and mortality rates, are key indicators for assessing the prognosis of stroke patients (6). Research has found that the mortality rate for acute ischemic stroke patients within 3 months is approximately 20 to 30% (7), while the proportion of functional recovery varies depending on the patients’ clinical characteristics and treatment measures.

Mean corpuscular volume (MCV) refers to the average volume of red blood cells and is typically used to assess the type of anemia and the health status of red blood cells (8). The normal range for MCV is typically 80–100 fL (9). Recent studies have shown that MCV is associated not only with anemia but also with the incidence and poor prognosis of various diseases. For example, elevated MCV has been found to be associated with increased mortality in patients with cardiovascular diseases (10). Additionally, changes in MCV are considered potential biomarkers for poor prognosis in various clinical conditions (11).

According to the existing literature, there is some controversy regarding the relationship between mean corpuscular volume (MCV) and 3 month outcomes in stroke patients. Some studies have shown that MCV is associated with the prognosis of stroke patients (12). For instance, research has found that MCV is related to functional outcomes in stroke patients (13). However, other studies have failed to find a significant association between MCV and 3 month outcomes, or the results are inconsistent, potentially influenced by sample size, study design, or other confounding factors (14). Importantly, while several studies have examined linear associations between MCV and stroke prognosis, the potential non-linear relationship—such as threshold or U-shaped patterns—has not been adequately explored. Therefore, this study aimed to investigate the non-linear association between MCV and 3 month functional outcomes in patients with acute ischemic stroke through a secondary analysis based on a prospective cohort, thereby providing new insights into its prognostic significance.

## Methods

### Study population

**Figure 1:**
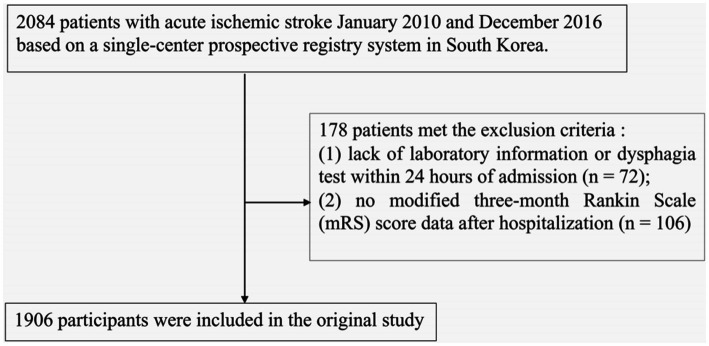
Flow chart of study population.

This study is a secondary analysis based on a previously published prospective cohort study conducted at a single tertiary center in South Korea from January 2010 to December 2016, involving patients with acute ischemic stroke who were admitted within 7 days of the study period. A total of 1,906 patients were included in the analysis. Exclusion criteria included a lack of laboratory information or swallowing function assessment within 24 h of admission, as well as missing 3 month mRS score data. Patients who did not undergo swallowing function assessment were excluded, as early swallowing function evaluation may impact the prevention of complications and prognosis. The flow chart of the study is shown in Figure 1.

### Data source

This study utilized data from a previously published research by Kang et al. (15), which investigated the relationship between geriatric nutritional risk index and outcomes in acute ischemic stroke patients. The original dataset was accessed through an open-access publication under the Creative Commons Attribution License. The source study was conducted at a single tertiary hospital in South Korea. Data can be downloaded from the ‘PLos one’ database.^1^

### Variables

The exposure variable in this study was mean corpuscular volume (MCV), measured using an automated blood cell analyzer within 24 h of admission. The outcome variable was the 3 month functional outcome, determined by the modified Rankin Scale (mRS) score. Covariates included age, sex, body mass index (BMI), hemoglobin, hematocrit, aspartate aminotransferase, blood urea nitrogen, albumin, fibrinogen, diabetes, history of stroke or transient ischemic attack, hypertension, coronary heart disease, stroke etiology, smoking history, and NIHSS score. Univariate logistic regression was first performed to identify variables potentially associated with the 3 month outcome (*p* < 0.10). These variables, together with clinically important factors reported in previous studies (e.g., age, sex, NIHSS score), were included in the multivariate model to control for confounding. Covariates were chosen based on clinical relevance and prior evidence linking them to both MCV and stroke prognosis. Missing data were minimal; triglyceride (TG) values were missing for 9 participants (<0.5%), and no missing values were observed for other variables. Thus, the influence of missing data on the overall results is likely negligible.

### Outcomes

Functional outcomes were assessed using the modified Rankin Scale (mRS) at 3 months after acute ischemic stroke onset. Outcome data were obtained through outpatient clinical visits or standardized telephone interviews. Based on the mRS scores, patients were dichotomized into two groups: favorable outcome (mRS 0–2) and unfavorable outcome (mRS 3–6).

### Statistical analysis

Continuous variables are presented as mean ± standard deviation or median (interquartile range), and categorical variables as numbers (percentages). Patients were stratified into quartiles based on mean corpuscular volume levels. One-way analysis of variance (ANOVA) was used to compare continuous variables among groups, while categorical variables were compared using chi-square tests. Generalized additive models (GAM) were employed to explore the dose–response relationship between mean corpuscular volume and outcomes. Logistic regression models were used to evaluate the association between mean corpuscular volume and 3 month outcomes, adjusting for confounding factors including age, sex, BMI, laboratory parameters (hemoglobin, hematocrit, AST, BUN, albumin, fibrinogen), underlying conditions (diabetes, previous stroke/TIA, hypertension, coronary heart disease), and clinical characteristics (stroke etiology, smoking status, NIHSS score).

A two-piecewise linear regression model was applied to examine the threshold effect between mean corpuscular volume and outcomes. The turning point was determined through “exploratory” analysis by moving the trial turning point along a pre-defined interval and selecting the point that yielded the maximum model likelihood. Log-likelihood ratio tests were performed to compare the fitness of one-line linear regression models with two-piecewise linear regression models. All statistical analyses were performed using R software version 4.0.3.^2^ A two-sided *p* < 0.05 was considered statistically significant.

Additionally, to assess the robustness of the observed associations to potential unmeasured confounding, E-values were calculated for the main odds ratios according to the method proposed by Vander Weele and Ding (16).

### Ethics statement

This study was a secondary analysis based on a previous prospective cohort study conducted at Seoul National University Hospital. All methods were performed in accordance with the relevant guidelines and regulations. The original study was approved by the Institutional Review Board of Seoul National University Hospital, and the Institutional Review Board waived the need for informed consent (IRB No. 1009–062-332).

## Results

### Characteristics of participants

Based on an analysis of 1,906 participants stratified into four quartiles by Mean Corpuscular Volume (MCV), we identified significant differences in demographic, metabolic, and clinical characteristics (most comparisons *p* < 0.001). Notable age-related trends were observed, with the proportion of participants under 60 years decreasing from 32.20 to 15.91%, while the proportion aged 70–80 years increased from 29.87 to 40.29% (*p* < 0.001). As MCV increased, the Body Mass Index (BMI) showed a significant decline from 23.70 ± 3.22 to 22.93 ± 3.11 (*p* < 0.001). Additionally, lipid profile alterations included a decrease in Total Cholesterol (TC) from 181.71 ± 46.95 mg/dL to 173.49 ± 42.15 mg/dL (*p* = 0.010), an increase in High-Density Lipoprotein Cholesterol (HDL-C) from 41.65 ± 16.60 mg/dL to 46.37 ± 16.87 mg/dL (*p* < 0.001), and a decline in Low-Density Lipoprotein Cholesterol (LDL-C) from 106.58 ± 45.30 mg/dL to 98.88 ± 38.58 mg/dL (*p* = 0.011). The prevalence of Diabetes Mellitus decreased from 42.16 to 27.69% (*p* < 0.001), while Atrial Fibrillation increased from 16.53 to 29.96% (*p* < 0.001). Stroke etiology also changed, with the proportion of Large Artery Atherosclerosis (LAA) decreasing from 37.08 to 26.45% and Cardioembolism increasing from 20.13 to 34.71% (*p* < 0.001). These findings suggest a complex interplay between MCV levels and age-related metabolic and cardiovascular modifications, indicating that MCV may serve as a potential biomarker reflecting systemic physiological changes across different life stages (Table 1).

**Table 1:** Baseline characteristics and 3 month outcomes according to quartiles of mean corpuscular volume (MCV) (*n* = 1,906).

Parameters	MCV (fl)				*p*-value
	Quartile 1	Quartile 2	Quartile 3	Quartile 4	
	65.9–89.8	89.9–92.8	92.9–95.9	96.0–121.5	
	*n* = 472	*n* = 481	*n* = 469	*n* = 484	
Continuous					
BMI (kg/m^2^), mean ± sd	23.70 ± 3.22	23.95 ± 3.39	23.41 ± 3.21	22.93 ± 3.11	<0.001
HGB (g/dL), mean ± sd	13.34 ± 2.14	13.72 ± 1.89	13.55 ± 1.95	13.31 ± 2.01	0.004
HCT (%), mean ± sd	39.53 ± 5.75	40.66 ± 5.28	40.30 ± 5.56	39.80 ± 5.72	0.009
PLT (10^9/L), mean ± sd	240.38 ± 81.60	231.16 ± 66.28	219.43 ± 67.84	203.81 ± 63.22	<0.001
TC (mg/dL), mean ± sd	181.71 ± 46.95	181.52 ± 42.75	180.44 ± 43.47	173.49 ± 42.15	0.010
TG^*^ (mg/dL), mean ± sd	108.72 ± 66.74	110.82 ± 61.36	101.73 ± 55.56	100.12 ± 55.01	0.013
HDL-c (mg/dL), mean ± sd	41.65 ± 16.60	43.81 ± 15.58	44.77 ± 17.83	46.37 ± 16.87	<0.001
LDL-c (mg/dL), mean ± sd	106.58 ± 45.30	106.89 ± 41.76	104.35 ± 43.41	98.88 ± 38.58	0.011
BUN (mg/dL),median (quartile)	15.00 (12.00–19.00)	15.00 (12.00–19.00)	16.00 (13.00–19.00)	16.50 (13.00–21.00)	0.006
Scr (mg/dL), median (quartile)	0.88 (0.73–1.09)	0.88 (0.73–1.07)	0.89 (0.73–1.05)	0.91 (0.76–1.12)	0.065
AST (U/L), median (quartile)	22.00 (17.00–28.00)	23.00 (18.00–29.00)	23.00 (19.00–29.00)	24.00 (20.00–31.00)	<0.001
ALT (U/L), median (quartile)	18.00 (13.00–28.00)	19.00 (14.00–26.00)	18.00 (14.00–25.00)	18.00 (13.00–26.00)	0.473
ALB (g/dL), mean ± sd	4.05 ± 0.46	4.05 ± 0.43	4.02 ± 0.41	3.95 ± 0.41	0.001
HBA1c (%), mean ± sd	5.29 ± 3.00	5.09 ± 2.77	5.17 ± 2.57	4.83 ± 2.64	0.071
FIB (mg/L), mean ± sd	337.16 ± 97.17	329.17 ± 91.82	330.56 ± 87.60	324.60 ± 90.73	0.206
NIHSS score, median (quartile)	3.00 (1.00–7.00)	3.00 (1.00–7.00)	3.00 (1.00–7.00)	4.00 (1.00–8.00)	0.290
Categorical					
Sex, *n* (%)					0.865
Male	290 (61.44%)	294 (61.12%)	281 (59.91%)	303 (62.60%)	
Female	182 (38.56%)	187 (38.88%)	188 (40.09%)	181 (37.40%)	
Age (years), *n* (%)					<0.001
<60	152 (32.20%)	114 (23.70%)	93 (19.83%)	77 (15.91%)	
60 to <70	115 (24.36%)	140 (29.11%)	126 (26.87%)	124 (25.62%)	
70 to <80	141 (29.87%)	162 (33.68%)	172 (36.67%)	195 (40.29%)	
≥80	64 (13.56%)	65 (13.51%)	78 (16.63%)	88 (18.18%)	
Previous stroke/TIA, *n* (%)	107 (22.67%)	94 (19.54%)	90 (19.19%)	111 (22.93%)	0.333
Hypertension, *n* (%)	307 (65.04%)	314 (65.28%)	294 (62.69%)	296 (61.16%)	0.489
Diabetes, *n* (%)	199 (42.16%)	160 (33.26%)	121 (25.80%)	134 (27.69%)	<0.001
Smoking, *n* (%)	173 (36.65%)	207 (43.04%)	184 (39.23%)	186 (38.43%)	0.226
Atrial fibrillation, *n* (%)	78 (16.53%)	93 (19.33%)	91 (19.40%)	145 (29.96%)	<0.001
CHD, *n* (%)	53 (11.23%)	54 (11.23%)	43 (9.17%)	70 (14.46%)	0.081
Stroke etiology, *n* (%)					<0.001
LAA	175 (37.08%)	160 (33.26%)	144 (30.70%)	128 (26.45%)	
SVO	82 (17.37%)	102 (21.21%)	93 (19.83%)	88 (18.18%)	
CE	95 (20.13%)	108 (22.45%)	122 (26.01%)	168 (34.71%)	
Other determined	57 (12.08%)	38 (7.90%)	36 (7.68%)	40 (8.26%)	
Undetermined	63 (13.35%)	73 (15.18%)	74 (15.78%)	60 (12.40%)	
The unfavorable outcome	99 (29.46%)	85 (22.79%)	84 (24.14%)	91 (26.69%)	0.189

Data are presented as mean ± standard deviation (SD), median (interquartile range), or percentage, as appropriate. ^*^Triglyceride (TG) data were missing for 9 participants (<0.5%), while no missing values were observed for other variables.

MCV, mean corpuscular volume; BMI, body mass index; HGB, hemoglobin; HCT, hematocrit; PLT, platelet count; TC, total cholesterol; TG, triglyceride; HDL-C, high-density lipoprotein cholesterol; LDL-C, low-density lipoprotein cholesterol; BUN, blood urea nitrogen; Scr, serum creatinine; AST, aspartate aminotransferase; ALT, alanine aminotransferase; ALB, albumin; FIB, fibrinogen; NIHSS, National Institutes of Health Stroke Scale; CHD, coronary heart disease; TIA, transient ischemic attack.

### Unadjusted association between baseline variables and the unfavorable outcomes

In this comprehensive univariate analysis of stroke patients, we systematically evaluated the impact of various demographic, clinical, and biological factors on stroke unfavorable outcomes. The results revealed several significant risk and protective factors (Table 2).

**Table 2:** The unadjusted association between baseline variables and the unfavorable outcome (*n* = 1906).

Parameters	Statistics	Odds ratio (95% CI)	*p* value
MCV (fl)	92.96 ± 5.22	0.99 (0.97, 1.01)	0.2187
MCV (fl) quartile			
Q1	472 (24.76%)	Reference	
Q2	481 (25.24%)	0.68 (0.51, 0.90)	0.0074
Q3	469 (24.61%)	0.74 (0.56, 0.97)	0.0323
Q4	484 (25.39%)	0.86 (0.65, 1.13)	0.2720
Sex, *n* (%)			
Male	1,168 (61.28%)	Reference	
Female	738 (38.72%)	1.66 (1.36, 2.03)	<0.0001
Age (years), *n* (%)			
<60	436 (22.88%)	Reference	
60 to <70	505 (26.50%)	1.15 (0.84, 1.59)	0.3801
70 to <80	670 (35.15%)	1.90 (1.42, 2.54)	<0.0001
≥80	295 (15.48%)	4.00 (2.87, 5.56)	<0.0001
BMI (kg/m^2^), mean ± sd	23.50 ± 3.25	0.92 (0.89, 0.94)	<0.0001
HGB (g/dL), mean ± sd	13.48 ± 2.00	0.82 (0.78, 0.86)	<0.0001
HCT (%), mean ± sd	40.07 ± 5.59	0.93 (0.92, 0.95)	<0.0001
PLT (10^9/L), mean ± sd	223.61 ± 71.31	1.00 (1.00, 1.00)	0.3042
TC (mg/dL), mean ± sd	179.26 ± 43.95	1.00 (0.99, 1.00)	0.0002
TG (mg/dL), mean ± sd	105.34 ± 59.97	1.00 (0.99, 1.00)	<0.0001
HDL-c (mg/dL), mean ± sd	44.16 ± 16.81	0.99 (0.99, 1.00)	0.0331
LDL-c (mg/dL), mean ± sd	104.15 ± 42.40	1.00 (0.99, 1.00)	0.0011
BUN (mg/dL), median (quartile)	17.60 ± 8.88	1.02 (1.01, 1.03)	0.0027
Scr (mg/dL), median (quartile)	1.09 ± 1.04	1.02 (0.93, 1.12)	0.7316
AST (U/L), median (quartile)	26.11 ± 14.34	1.01 (1.00, 1.02)	0.0086
ALT (U/L), median (quartile)	22.38 ± 16.10	0.99 (0.99, 1.00)	0.0409
ALB (g/dL), mean ± sd	4.02 ± 0.43	0.27 (0.21, 0.35)	<0.0001
HBA1c (%), mean ± sd	5.09 ± 2.75	1.01 (0.97, 1.05)	0.6481
FIB (mg/L), mean ± sd	330.33 ± 91.93	1.00 (1.00, 1.00)	<0.0001
NIHSS score, median (quartile)	5.39 ± 5.72	1.23 (1.20, 1.26)	<0.0001
Previous stroke/TIA, *n* (%)			
No	1,504 (78.91%)	Reference	
Yes	402 (21.09%)	1.81 (1.44, 2.28)	<0.0001
Hypertension, *n* (%)			
No	695 (36.46%)	Reference	
Yes	1,211 (63.54%)	1.34 (1.09, 1.66)	0.0061
Diabetes, *n* (%)			
No	1,292 (67.79%)	Reference	
Yes	614 (32.21%)	1.45 (1.17, 1.78)	0.0005
Smoking, *n* (%)			
No	1,156 (60.65%)	1.0	
Yes	750 (39.35%)	0.61 (0.49, 0.75)	<0.0001
Atrial fibrillation, *n* (%)			
No	1,499 (78.65%)	Reference	
Yes	407 (21.35%)	2.00 (1.59, 2.52)	<0.0001
CHD, *n* (%)			
No	1,686 (88.46%)	Reference	
Yes	220 (11.54%)	1.02 (0.75, 1.40)	0.8768
Stroke etiology, *n* (%)			
LAA	607 (31.85%)	Reference	
SVO	365 (19.15%)	0.62 (0.45, 0.85)	0.0031
CE	493 (25.87%)	1.49 (1.15, 1.92)	0.0025
Other determined	171 (8.97%)	2.06 (1.45, 2.93)	<0.0001
Undetermined	270 (14.17%)	0.86 (0.61, 1.19)	0.3598

Data are expressed as mean ± SD, median (interquartile range), or percentage.

Gender analysis demonstrated that female patients have a significantly higher stroke unfavorable outcome risk compared to males (OR = 1.66, 95% CI: 1.36–2.03, *p* < 0.0001). Age emerged as a critical influencing factor, with particularly pronounced risk in populations over 70 years, where the stroke unfavorable outcome risk significantly increases (≥80 years group: OR = 4.00, 95% CI: 2.87–5.56, *p* < 0.0001) (Table 2).

Previous stroke/TIA history (OR = 1.81, 95% CI: 1.44–2.28, *p* < 0.0001) and atrial fibrillation (OR = 2.00, 95% CI: 1.59–2.52, *p* < 0.0001) substantially elevated stroke unfavorable outcomes risk. In the stroke etiology analysis, Cardiogenic Embolism (CE) and other determined cause groups showed 1.49-fold (95% CI: 1.15–1.92, *p* = 0.0025) and 2.06-fold (95% CI: 1.45–2.93, *p* < 0.0001) increased risks, respectively (Table 2).

Neurological deficit severity (NIHSS) demonstrated a significant positive correlation with stroke unfavorable outcome risk, with a 23% risk increase per point (OR = 1.23, 95% CI: 1.20–1.26, *p* < 0.0001). Notably, albumin levels exhibited a significant protective effect, with a 73% risk reduction per unit increase (OR = 0.27, 95% CI: 0.21–0.35, *p* < 0.0001) (Table 2).

### Identification of nonlinear relationship

**Figure 2:**
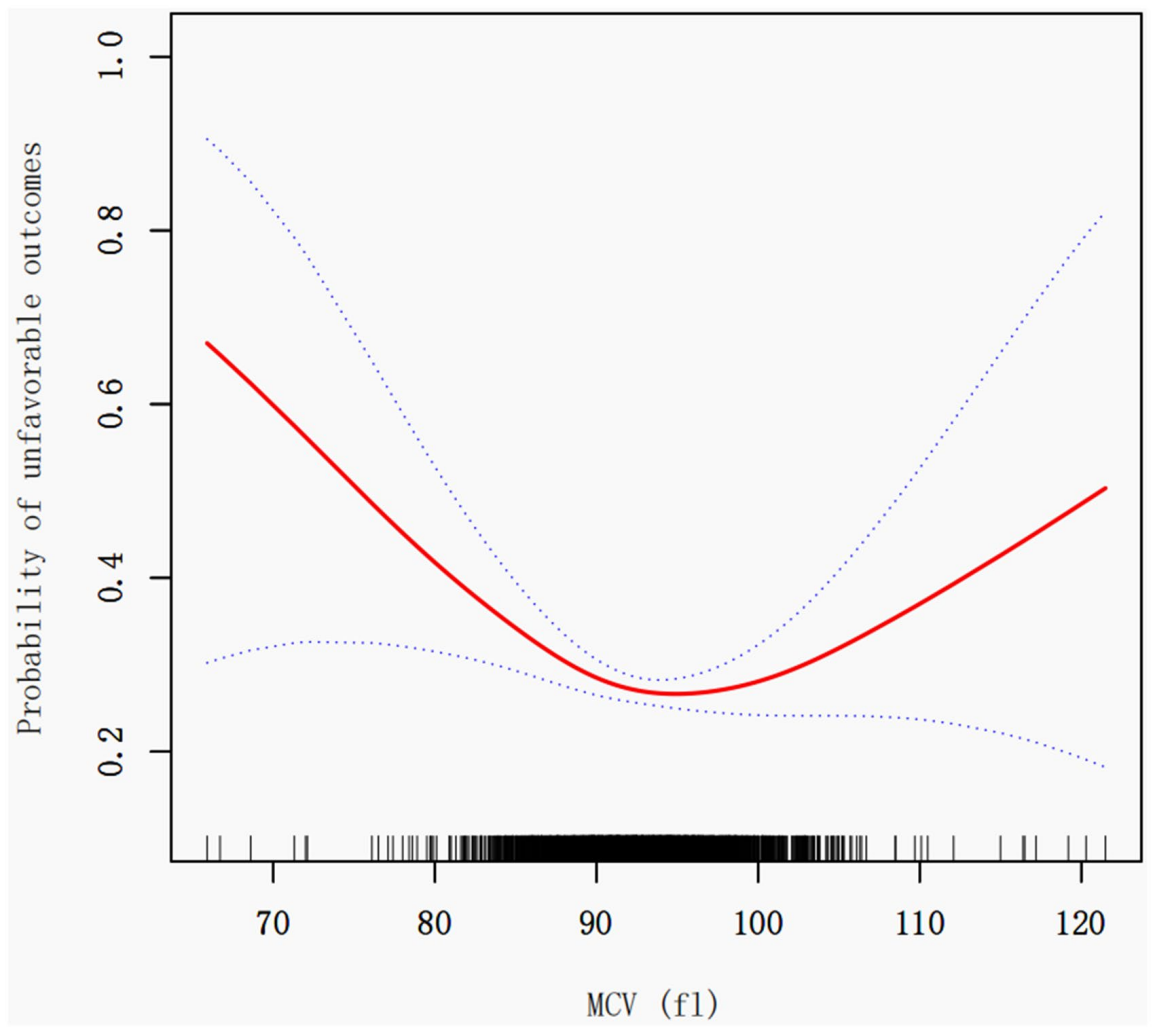
Association between MCV and the unfavorable outcome in patients with acute ischemic stroke. A threshold, nonlinear association between the MCV and 3 month outcome was found in a generalized additive model (GAM). The red solid line represents the smooth curve fit between variables. The blue bands represent the 95% confidence interval from the fit. Adjusted for age, sex, BMI, laboratory parameters (hemoglobin, hematocrit, AST, BUN, albumin, fibrinogen), underlying conditions (diabetes, previous stroke/TIA, hypertension, coronary heart disease), and clinical characteristics (stroke etiology, smoking status, NIHSS score).

In investigating the impact of Mean Corpuscular Volume (MCV) on stroke unfavorable outcomes, we employed an innovative piecewise linear regression model, revealing the complex non-linear relationship between MCV and adverse clinical results (Figure 2; Table 3).

**Table 3:** Threshold effect analysis of MCV and the unfavorable outcome.

Unfavorable outcome	Model I		Model II		Model III	
	OR (95%CI)	*p* value	OR (95%CI)	*p* value	OR (95%CI)	*p* value
Fitting model by standard linear regression						
One line effect	0.99 (0.97, 1.01)	0.2187	0.98 (0.96, 1.00)	0.0143	0.99 (0.96, 1.01)	0.2260
Fitting model by two-piecewise linear regression						
Turning point (K)	92.1		92.3		92.1	
MCV < K	0.92 (0.89, 0.95)	<0.0001	0.91 (0.88, 0.95)	<0.0001	0.92 (0.88, 0.96)	0.0003
MCV > K	1.04 (1.01, 1.08)	0.0052	1.03 (1.00, 1.07)	0.0466	1.04 (1.00, 1.08)	0.0391
P value for LRT test*		<0.001		<0.001		<0.001

Data are presented as odds ratios (OR) (95% confidence interval) and *p* values; Model I is not adjusted. Model II adjusted for age, sex, BMI. Model III adjusted for age, sex, BMI, HGB, HCT, AST, BUN, ALB, FIB, diabetes, previous stroke/TIA, hypertension, CHD, stroke etiology, smoking, NIHSS score. CI stands for confidence interval, OR stands for odds ratio, and LRT stands for likelihood ratio test. *p < 0.05 indicates that fitting model by two-piecewise linear regression is significantly different from fitting model by standard linear regression.

Through rigorous statistical analysis, we identified a critical MCV threshold point at 92.1 fl. Notably, the effect of MCV on unfavorable outcomes demonstrates significant variations before and after this threshold. Each 1 fL increase in MCV (when MCV < 92.1 fL) was associated with an 8% lower odds of poor 3 month outcome (OR = 0.92, 95% CI: 0.89–0.95, *p* < 0.0001). Conversely, when MCV exceeds 92.1 fl, the risk of unfavorable outcomes slightly increases [OR = 1.04, 95% CI: 1.01–1.08, *p* = 0.0052], with each unit increase in MCV resulting in a approximately 4% higher risk (Table 3).

The Likelihood Ratio Test (LRT) results (*p* < 0.001) provide robust support for the piecewise linear model’s significance, conclusively demonstrating the non-linear threshold effect between MCV and unfavorable outcomes.

To further evaluate the robustness of these findings to potential unmeasured confounding, E-values were calculated. The E-value for MCV < 92.1 fL was 1.43 and for MCV > 92.1 fL was 1.29, indicating moderate robustness to residual confounding.

In a sensitivity analysis excluding patients with baseline anemia (defined as hemoglobin <12 g/dL for women and <13 g/dL for men), 1,398 participants remained. The results (see Supplementary Table S1) were consistent with the main findings, confirming a persistent U-shaped association between MCV and 3 month unfavorable outcomes.

## Discussion

In this cohort study involving 1,906 patients, the research innovatively employed a piecewise linear regression model to explore the U-shaped relationship between MCV and stroke prognosis. A critical threshold of 92.1 fl was identified: Each 1 fL increase in MCV (when MCV < 92.1 fL) was associated with an 8% lower odds of poor 3 month outcome (OR = 0.92, 95% CI: 0.89–0.95, *p* < 0.0001); conversely, when MCV exceeded 92.1 fl, each unit increase slightly elevated unfavorable outcome risk by about 4% (OR = 1.04, 95% CI: 1.01–1.08, *p* = 0.0052). To illustrate the clinical significance of the U-shaped relationship, we compared the absolute risk of unfavorable outcomes (mRS ≥ 3) across MCV quartiles. Patients at the lowest (Q1, 87.1 fL) and highest (Q4, 98.8 fL) quartiles had 6.7 and 3.9% higher risks of unfavorable outcomes compared with those near the threshold (Q2, 91.4 fL), respectively. This finding underscores that even modest deviations in MCV within the normal range may have prognostic implications.

MCV is a parameter in the complete blood count (CBC) used for the clinical differential diagnosis of anemia. Currently, only a few clinical studies have assessed the association between MCV and outcomes in cerebrovascular diseases. Zhang et al. (17) analyzed the relationship between Mean Corpuscular Volume (MCV) and 30-day mortality in intracerebral hemorrhage patients using the MIMIC-III database, finding significantly higher mortality in the high MCV group (≥92 fl). Wu et al. (18) further supported the association between MCV and disease prognosis, discovering a gradient relationship between MCV levels and mortality in ischemic stroke and ischemic heart disease among 66,294 Taiwanese individuals. Wang et al. (19) highlighted the close association between MCV and post-stroke psychiatric disorders. Gamaldo et al. (20) found that cognitive recovery in early post-stroke cognitive impairment survivors was related to MCV levels. Overall, although some studies support the association between MCV and outcomes in stroke patients, the diversity of research findings and methodological differences have prevented a clear consensus from forming. Therefore, it can be said that the research results in this field are controversial.

We observed a U-shaped association between MCV and 3 month outcomes in patients with acute ischemic stroke. The potential mechanisms underlying this relationship may involve multiple pathophysiological pathways. Several hypotheses have been proposed regarding how both elevated and decreased MCV levels might contribute to adverse cerebrovascular outcomes. First, larger red blood cells (RBCs) may become obstructed within the microcirculation, potentially increasing blood viscosity and reducing cerebral perfusion, which could exacerbate ischemic injury and lead to poorer functional outcomes (21). Secondly, higher MCV levels might reflect underlying metabolic or nutritional imbalances, such as iron or folate deficiency, low body mass index, low LDL cholesterol, or elevated homocysteine, all of which are associated with increased cerebrovascular risk (22). Third, the body’s antioxidant capacity may be linked to the total circulating RBC volume (23). thus, an increase in MCV could be associated with alterations in oxidative balance, which may partially explain the observed relationship. Conversely, a lower MCV is often observed in inflammatory states. In such conditions, elevated hepcidin levels can interfere with iron metabolism, reducing erythropoiesis, while increased IL-6 concentrations may suppress erythroid precursor proliferation, thereby decreasing MCV values (24). In addition, lower MCV levels have been associated with elevated thrombomodulin and activation of thrombin-activatable fibrinolysis inhibitor (TAFI), which may reduce fibrinolytic activity and increase thrombosis risk (25). Finally, microcytic anemia (MCV < 80 fL)—often related to iron deficiency or hemolytic anemia—could also partly explain the poorer prognosis observed among individuals with lower MCV values (26). Taken together, these hypotheses suggest possible biological pathways linking MCV to stroke outcomes; however, causal relationships cannot be established based on this observational study, and further experimental and longitudinal research is warranted to confirm these mechanisms.

Our study innovatively used a piecewise linear regression model to systematically reveal the non-linear U-shaped association between MCV and adverse outcomes, with a critical turning point at 92.1 fl. Compared to existing research, our methodology is more refined, not only quantifying MCV’s impact on prognosis but also elucidating its complex non-linear mechanism. The identified threshold of 92.1 fL lies within the conventional normal MCV range (80–100 fL), but closer to its lower limit, suggesting that even subtle reductions in MCV within the ‘normal’ range may carry prognostic significance. Previous studies support this interpretation: for instance, Li et al. (27) observed nonlinear relationships between MCV and cardiovascular mortality in a U.S. cohort; Wu et al. (18) reported that MCV is positively associated with vascular mortality in a Taiwanese population; and Wang et al. (28) found that MCV correlates with the severity of coronary artery disease. Therefore, the 92.1 fL cutoff identified in this study is clinically intuitive and highlights that mild microcytic tendencies may reflect subclinical inflammation or impaired erythropoiesis that adversely affect stroke recovery.

The primary strengths of our study lie in its innovative research design and rigorous data analysis strategies. First, we utilized data from a previously published Korean prospective cohort of acute ischemic stroke patients, which substantially enhanced the study’s sample size and representativeness. Second, we employed Logistic regression model, progressively adjusting for potential confounding factors such as age, gender, body mass index, hemoglobin, hematocrit, liver function indicators, diabetes history, and stroke etiology, substantially improving the internal validity of the results. Although this study did not include *a priori* sample size calculation, a post-hoc power analysis indicated that with a total of 1,906 participants and an unfavorable outcome rate of approximately 25%, the study had more than 90% statistical power to detect an association of the observed magnitude (OR = 0.92) at a two-sided *α* of 0.05. Therefore, the sample size was sufficient to ensure the reliability of the main findings.

Several inevitable limitations of this study warrant acknowledgment. First, this was a secondary analysis based on a single-center Korean prospective cohort study, which may limit the generalizability and external validity of our findings. Differences in ethnicity, dietary patterns, clinical management practices, and healthcare systems may influence MCV levels and stroke outcomes. Therefore, multi-center studies involving diverse populations are needed to confirm the external validity of these results. Second, despite the large sample size, caution is advised when applying our findings to other racial or geographical populations. Additionally, the inclusion criterion requiring completion of a swallowing function test may have introduced selection bias by excluding patients with severe neurological deficits or unstable conditions who were unable to undergo such testing. Consequently, the study population may not fully represent the entire spectrum of patients with acute ischemic stroke, and our findings should be interpreted with caution when extrapolated to broader populations. Third, as an observational study, despite employing rigorous statistical methods, we can only demonstrate an association between MCV and stroke prognosis rather than a causal relationship. Fourth, although we adjusted for multiple potential confounding factors, unmeasured confounders such as genetic background and lifestyle habits may still have influenced the results. Lastly, our study did not explore the underlying biological mechanisms driving the U-shaped association between MCV and outcomes, which should be addressed in future research.

## Conclusion

In conclusion, a U-shaped association was observed between MCV and 3 month outcomes in patients with acute ischemic stroke. MCV may serve as a potential prognostic biomarker to assist in risk stratification. However, as this study was based on a single-center Korean cohort, caution should be exercised when generalizing these findings to other populations. Validation in multicenter and multiethnic cohorts is warranted to confirm the robustness and applicability of our results.

## Data Availability

The datasets presented in this study can be found in online repositories. The names of the repository/repositories and accession number(s) can be found in the article/Supplementary material.

## Footnotes

^1^ https://doi.org/10.1371/journal.pone.0228738.s001

^2^ http://www.r-project.org

## Supplementary material

SUPPLEMENTARY TABLE S1 Threshold effect analysis of mean corpuscular volume (MCV) and 3 month unfavorable outcomes in non-anemic patients with acute ischemic stroke. Data are presented as odds ratios (OR) (95% confidence interval) and *p* values; Model I is not adjusted. Model II adjusted for age, sex, BMI. Model III adjusted for age, sex, BMI, HGB, HCT, AST, BUN, ALB, FIB, diabetes, previous stroke/TIA, hypertension, CHD, stroke etiology, smoking, NIHSS score. CI stands for confidence interval, OR stands for odds ratio, and LRT stands for likelihood ratio test. **p* < 0.05 indicates that fitting model by two-piecewise linear regression is significantly different from fitting model by standard linear regression.
